# The association of *Streptococcus bovis/gallolyticus *with colorectal tumors: The nature and the underlying mechanisms of its etiological role

**DOI:** 10.1186/1756-9966-30-11

**Published:** 2011-01-20

**Authors:** Ahmed S Abdulamir, Rand R Hafidh, Fatimah Abu Bakar

**Affiliations:** 1 Institute of Bioscience, University Putra Malaysia, 43400 Serdang, Selangor, Malaysia

## Abstract

*Streptococcus bovis *(*S. bovis*) bacteria are associated with colorectal cancer and adenoma. *S. bovis *is currently named *S. gallolyticus*. 25 to 80% of patients with *S. bovis/gallolyticus *bacteremia have concomitant colorectal tumors. Colonic neoplasia may arise years after the presentation of bacteremia or infectious endocarditis of *S. bovis/gallolyticus*. The presence of *S. bovis/gallolyticus *bacteremia and/or endocarditis is also related to the presence of villous or tubular-villous adenomas in the large intestine. In addition, serological relationship of *S. gallolyticus *with colorectal tumors and direct colonization of *S. gallolyticus *in tissues of colorectal tumors were found. However, this association is still under controversy and has long been underestimated. Moreover, the etiological versus non-etiological nature of this associationis not settled yet. Therefore, by covering the most of up to date studies, this review attempts to clarify the nature and the core of *S. bovis/gallolyicus *association with colorectal tumors and analyze the possible underlying mechanisms.

## Introduction & statement of the problem

One of the bacterial agents that has been found to be regularly associated with colorectal cancer is *Streptococcus bovis *(*S. bovis*). *S. bovis *has been shown to have important impact on health since 25 to 80% of patients with *S. bovis *bacteremia have colorectal tumors and the incidence of association of colonic neoplasia with *S. bovis *endocarditis has been shown to be 18 to 62% [1-7]. It was shown that 94% of *S. bovis *bacteremia associated with colorectal cancer was in fact *S. bovis *biotype I while only 18% was associated with biotype II [8]. Later, a new species resembling *S. bovis *was detected which was named *S. gallolyticus *[9]. Interestingly, *S. bovis *biotype I and II/2 isolates were then found to be *S. gallolyticus *[10]. Accordingly, *S. bovis *biotype I was renamed as *S. gallolyticus *subspecies *gallolyticus *and biotype II/2 was renamed as *S. gallolyticus *subspecies *pasterianus *and *S. gallolyticus *subspecies *macedonicus *[11] (Table 1). *S. gallolyticus *subspecies *gallolyticus *bacteria, more than other related taxa, have been found to be constantly associated with underlying colorectal cancer [10]. Therefore, the term *S. bovis/gallolyticus *is used in the current review.

**Table 1:** The milestone of the taxonomy of *S. bovis/gallolyticus *and the closely related members of group D streptococci [11,127].

Old nomenclature	Later nomenclature	Recent nomenclature
*S. bovis *biotype I	*S. gallolyticus*	*S. gallolyticus *subsp. *gallolyticus*
*S. bovis *biotype II/1	*S. infantarius*	*S. infantarius *subsp. *infantarius*
	*S. infantarius *subsp. *Coli*	*S. lutetiensis*
*S. bovis *biotype II/2	*S. pasteurianus S. macedonicus*	*S. gallolyticus *subsp. *Pasteurianus S. gallolyticus subsp. macedonicus*

Unfortunately, the nature of the association between *S. bovis/gallolyticus *and colorectal cancer has long been underestimated. It has been controversial whether the association of *S. bovis/gallolyticus *bacteremia or endocarditis with colorectal tumors is merely a consequence of the gastrointestinal lesion or it could be of etiological nature. Furthermore, there is a growing need to highlight the possible mechanisms that *S. bovis/gallolyticus *might play in triggering or promoting colorectal cancer, if any. Moreover, the relationship of this bacterium with oncogenic factors, cell growth factors, and pro-inflammatory cytokines has not yet been clarified well. Therefore, the current review was done to scrutinize the nature and the underlying mechanisms of the association of *S. bovis/gallolyticus *with colorectal cancer.

## Bacterial pathogens and cancer

Traditionally, bacterial infections have not been considered a major cause of cancer. However, bacteria have been linked to cancer by two mechanisms: chronic inflammation and production of carcinogenic metabolites [12]. It was stated that bacteria in general are thought to contribute to carcinogenesis by the formation of potentially toxic by-products of carbohydrates or bile acid metabolism, as well as hydrolysis of other mutagenic precursors [12].

The association of *Helicobacter pylori *(*H. pylori*) with gastric cancer is the best studied relationship between a bacterial infection and cancer [13]. *H. pylori *has been recognized as a class I human gastric carcinogen by the International Agency for Research on Cancer [14]. The mechanisms by which bacteria contribute to cancer formation are complex and involve the interplay among chronic inflammation, direct microbial effects on host cell physiology, and changes in tissue stem cell homeostasis [15]. In fact, researchers in the field recently started to be sure that some chronic bacterial infections are associated with tumors formation; so, it might be possible to prevent or treat some forms of cancer if the infectious source was addressed [16].

A marked resurgence of interest in the gastrointestinal commensal flora and local host-microbe interactions was observed since it was recognized that intestinal bacteria could be implicated in the pathogenesis of several inflammatory diseases like Crohn's disease or ulcerative colitis [17]. Both diseases are commonly suspected to result from altered host responses to intestinal bacterial flora [18], and are associated with cancer risk [17,19-21]. Accordingly, World Health Organization considered bacteria as possible causative agents for cancer development.

## Colorectal cancer and infection

The incidence of colorectal cancer varies widely among countries. In the developed world, colorectal cancer represents a major public health problem. In the UK and the USA, colorectal cancer is the second most common cancer after breast cancer for women, and prostate or lung cancer for men [22-25].

The involvement of intestinal microflora in the pathogenesis of colon cancer has been hypothesized. Many cancers arise from sites of infection, chronic irritation, and inflammation [26]. The strongest association of chronic inflammation with malignant diseases is found in inflammatory bowel diseases of colon [27] with a lifetime incidence of 10% [28,29].

The gut is colonized by many species of bacteria, and it is nearly impossible to narrow carcinogenesis to one organism, but it is possible that a specific bacterium may cause a favorable microclimate for mutagens to inflict their damage [12]. Some studies provided evidence that some colorectal cancers might be caused by infectious agents. One group of researchers found that bacterial methyltransferases induce mutations in tumor suppressor genes [30]. Another group found that some microflora might serve as promoters while others might serve as anti-promoters of colorectal carcinogenesis [31]. A third group concentrated their studies on colicins, which were found to exert antitumor effects [32,33].

Later studies showed that cytokine-based sequel of long-standing bacterial inflammation might be the main mechanism of transformational changes in normal colorectal mucosa. In *H. pylori *infections, the gastric levels of cytokines were found to correlate strongly with inflammation and the degree of gastritis [21,34]. It was also reported that colonic cells exposed *in vitro *to *Clostridium difficile *toxin A showed induced cytokines production [35,36]. Alike, *S. bovis*/*gallolyticus *bacteria, especially their cell wall antigens, were found to increase remarkably the production of inflammatory cytokines in the colonic mucosa of rats, suggesting direct interaction between *S. bovis *and colonic mucosal cells which is thought to lead to the development of colorectal cancer [37-40]. Hence, collectively, the bacterial etiology/predisposition of colorectal cancer has become evidently prevailing in the field of research which necessates intensive evaluation of the current trend of research done in this field.

## The association of *S. bovis/gallolyticus *bacteremia/endocarditis with colorectal cancer

*S. bovis *was traditionally considered as a lower grade pathogen frequently involved in bacteremia and endocarditis. Although McCoy and Mason [41] suggested a relationship between colonic carcinoma and the presence of infectious endocarditis in 1951, it was only in 1974 that the association of *S. bovis *and colorectal neoplasia was recognized [42]. Nevertheless, the extent, nature, and basis of this association are still not completely understood. A recent study [43] sequenced the 2,350 Kb genome of *S. gallolyticus *and analyzed 2,239 encoded proteins; they found that this bacterium synthesizes many proteins and polysaccharides for the assembly of capsular sheath, collagen-binding proteins, and three types of pili that all render this bacterium highly efficient in causing bacteremia, endocarditis, and colorectal cancer.

The association of *S. bovis/gallolyticus *bacteremia/endocarditis with colorectal cancer was assessed by numerous studies. It was found that 25 to 80% of patients with *S. bovis/gallolyticus *bacteremia and 18 to 62% of patients with *S. bovis/gallolyticus *endocarditis have underlying colorectal tumors [1-7,44,45]. The high rate of this association indicates serious clinical impact given that *S. bovis/gallolyticus *accounts for 14% of the cases of infectious endocarditis, and 13% of all cases of infectious endocarditis are caused by bacteria of gastrointestinal origin [46]. A study conducted for 18 years in Spain showed increased incidence of infective endocarditis cases casued by *S. bovis/gallolyticus *indicating that *S. bovis*/gallolyticus bacteremia/endocarditis is an emergent disease [45]. Thorough studies on *S. bovis *showed that the association between *S. bovis *bacteraemia and carcinoma of the colon and infective endocarditis is biotype-specific. It was shown that there is 94% association between *S. bovis *biotype I bacteraemia and infective endocarditis and 71% association between *S. bovis *biotype I bacteraemia and colonic carcinoma while it is only 18% association between *S. bovis *biotype II bacteraemia and infective endocarditis and 17% association between *S. bovis *biotype II bacteraemia and colonic carcinoma [8]. Following the description of *S. gallolyticus*, Devriese team used whole-cell protein analysis showing that the bacterial isolates studied by his team, which were derived from patients with endocarditis and identified by conventional techniques as *S. bovis*, were in fact *S. gallolyticus*. Therefore, they suggested that *S. gallolyticus *is more likely to be involved in human infections than *S. bovis *[10].

The wide range of the association rates between *S. bovis/gallolyticus *and colorectal cancer might be attributed to different geographical and ethnic groups studied so far [47]. In a study conducted in Hong Kong, *S. bovis *biotype II/2 (*S. gallolyticus *subspecies *pasterianus*), rather than biotype I (*S. gallolyticus *subspecies *gallolyticus*), was found to be dominantly associated with colorectal tumors [48] while, in Europe and the USA, *S. gallolyticus *subspecies *gallolyticus *is dominantly associated with colorectal tumors [10,47].

Beside the characteristic adhesive traits of *S. bovis/gallolyticus *to the intestinal cells, it is also known that, in contrast to most α-haemolytic streptococci, *S. bovis/gallolyticus *is able to grow in bile [49] Therefore, unlike other bacteria, *S. bovis/gallolyticus *can bypass efficiently the hepatic reticulo-endothelial system and access systemic circulation easily which might explain the route responsible for the association between *S. bovis/gallolyticus *colonic lesions and *S. bovis/gallolyticus *bacteremia [50]. In this regard, an association was found between *S. bovis/gallolyticus *bacteraemia/endocarditis and liver disease [50]. The prevalence of chronic liver disease in patients with *S. bovis/gallolyticus *endocarditis was significantly higher than in patients with endocarditis caused by another aetiology (60% vs 15.3%) [51]. The rate of simultaneous occurrence of liver disease and colon cancer in patients with *S. bovis/gallolyticus *endocarditis/bacteraemia was found to be 27% [4]. Therefore, it was inferred that the association of *S. bovis/gallolyticus *bacteraemia/endocarditis with colorectal neoplasia indicates special pathogenic traits of this bacteria rendering it capable of entering blood circulation selectively through hepatic portal route. Accordingly, it was recommended that the liver as well as the bowel should be fully investigated in patients with *S. bovis/gallolyticus *endocarditis/bacteraemia [4,50-52]. Nevertheless, this does not exclude the possibility that other intestinal bacteria might be associated with colon cancer; a rare report stated that cases of *Klepsiella pneumoniae *liver abscess were found to be associated with colon cancer [53,54].

## The extra colonic affection of *S. bovis/gallolyticus *bacteria

Beside infective endocarditis, case reports suggested the possibility of infections by *S. bovis/gallolyticus *in various sites outside colorectum such as osteomyelitis, discitis [55] and neck abscess [56] which could be linked to colonic malignancy or malignancies in other locations. Although many studies suggested that infective endocarditis is the commonest manifestation of *S. bovis/gallolyticus *infection in western countries [5-7,50], cholecystitis, cholangitis, and biliary tract diseases were reported to be commonest manifestations in other geographical areas, such as Hong Kong [48].

In addition, it was found that *S. bovis/gallolyticus *bacteremia is associated with malignancy irrespective of site; 29% of patients with positive *S. bovis/gallolyticus *bacteremia harbored tumor lesions in the colon, duodenum, gallbladder, pancreas, ovary, uterus, lung, or hematopoietic system [57]. Moreover, other studies observed the occurrence of *S. bovis/gallolyticus *bacteremia in patients with pancreatic cancer [58,59], squamous cell carcinoma of the mouth [59,60], endometrial cancer [61], melanoma metastatic to the gastrointestinal tract [62], lymphosarcoma [63], Kaposi sarcoma [64], esophageal carcinoma [65], gastric carcinoma [66], gastric lymphoma [67] and pancreatic carcinoma [68].

## The association of *S. bovis/gallolyticus *with colorectal adenoma

High incidence of colorectal cancer in individuals with polyps was observed. Most cases of invasive colorectal adenocarcinomas were found to arise from pre-existing adenomatous polyps [69]. About 90% of preinvasive neoplastic lesions of the colorectum are polyps or polyp precursors, namely aberrant crypt foci [70]. Neoplastic polyps are often referred to more specifically as adenomas or adenomatous polyps [71]. Adenomatous polyps are considered as good and few surrogate end point markers for colorectal cancer [70,72].

It would be of interest to scrutinize any relationship between *S. bovis/gallolyticus *and colonic polyps taking into account the type of polyp and its malignant potential [11,47]. The relationship between *S. bovis/gallolyticus *infection and the progressive development of malignant disease in preneoplastic adenomatous polyps was supported by recent reports [39,73,74]. Interestingly, *S. bovis/gallolyticus *was found to be mildly associated with some benign lesions (diverticulosis, inflammatory bowel disease, cecal volvulus, perirectal abscess hemorrhoids, and benign polyps), while it was strongly associated with most malignant diseases (cancer and neoplastic polyps) of the colon [2,39,67,70,75,76]. It was also revealed that *S. bovis/gallolyticus *in patients with bacteremia and/or endocarditis is selectively related to the presence of the most aggressive type of polyps in the large intestine, villous or tubulovillous adenomas, [76,77] In addition, Hoen team performed a case-control study on subjects underwent colonoscopy comparing between patients with *S. bovis/gallolyticus *endocarditis and sex- and age- matched unaffected patients. This study showed that colonic adenomatous polyps in the patients' group were twice as many cases as controls (15 of 32 *vs *15 of 64), while lesions of colorectal cancer were present approximately 3 times as often as controls (3 of 32 *vs *2 of 64) [78]. On the other hand, another study [79] found that the association between *S. bovis/gallolyticus *and adenoma is more evident than colorectal cancer; they reported that 36% of positive blood cultures of *S. bovis/gallolyticus *were found in proliferative lesions, 15% of cancers and 21% of adenomas. A recent study done by our team supported this concept [39] showing that the level of *S. bovis/gallolyticus *IgG antibodies in adenoma patients was higher than in colorectal cancer patients or control subjects. However, Burns et al. [75] did not get the same findings; they found that the incidence of *S. bovis/gallolyticus *carriage in all colons with polyps was intermediary between normal colons and colons with carcinoma; however, the difference did not achieve statistical significance.

Since there is evidence that colon cancer progresses from normal tissue to adenoma and then to carcinoma through an accumulation of genetic alterations [80], the remarkable association between *S. bovis/gallolyticus *and adenomatous polyps seems to be of importance. Although ulceration of neoplastic lesions might form a pathway for *S. bovis/gallolyticus *to enter the bloodstream [7], the association of *S. bovis/gallolyticus *bacteremia with non-ulcerated colonic polyps indicates an etiological/promoter role of *S. bovis/gallolyticus *in polyps progression [81,82]. Therefore, the possibility of *S. bovis/gallolyticus *to act as a promoter for the preneoplastic lesions worths consideration. Ellmerich et al. [37] supported this hypothesis. They treated normal rats with *S. bovis *wall extracted antigens; rats did not develop hyperplastic colonic crypts; however, 50% of rats, that already received a chemocarcinogen, developed neoplastic lesions upon receiving *S. bovis *wall extracted antigens. This indicated that *S. bovis/gallolyticus *might exert their carcinogenic activity in colonic mucosa when preneoplastic lesions are established. Therefore, the role of *S. bovis/gallolyticus *in the etiology and/or acceleration of the transformation of aberrant crypts to adenoma and to a cancer is being considered.

Accordingly, the knowledge of *S. bovis/gallolyticus *association with adenoma of colorectal mucosa has important clinical implications. If colorectal lesions could be discovered at an early stage, curative resection might become possible [83]. Thus, bacteremia due to *S. bovis/gallolyticus *should prompt rigorous investigation to exclude both endocarditis and tumors of the large bowel [82,84]. Therefore, it was concluded that the discovery of a premalignant proliferative lesion in patients with history of bacteremia/endocarditis justifies the exploration of the colon by barium enema and/or colonoscopy [82,84].

## Etiological versus non-etiological role of *S. bovis/gallolyticus *in the development of colorectal tumors

The underlying mechanisms for the association of *S. bovis/gallolyticus *bacteremia/endocarditis with colorectal tumors have long been obscure. The possible reason behind that, maybe, *S. bovis/gallolyticus *is a member of intestinal flora in 2.5 to 15% of individuals; this usually leads scientists to counteract the malicious role of this bacteria [44,75]. Therefore, a big question is frequently asked whether *S. bovis/gallolyticus *plays an etiological role in the development of colorectal tumors or it is merely a marker of the disease.

There are many clues provide strong evidence for the etiological role of *S. bovis/gallolyticus *in colon cancer development. The striking association between bacteremia caused by *S. bovis *biotype I and both colonic neoplasia (71%) and bacterial endocarditis (94%), compared with bacteremias caused by the closely related organisms such as *S. bovis *variant and *S. salivarius*, suggests the possibility of specific bacterium-host cell interaction involving *S. bovis *biotype I organisms [85]. Later, *S. gallolyticus *subspecies *gallolyticus*, rather than other closely related taxa, was found to be actively colonizing colorectal tumors and primarily associated with colorectal cancer [40]. In addition, these bacteria showed special predilection to colonic lesions rather than other members of group D Streptococcus endocarditis. It was found that of 77 infections with group D Streptococcus endocarditis, colonic polyps and colonic carcinoma were significantly more frequent in the *S. bovis/gallolyticus *group, 67 and 18%, than in the Enterococcus group, 21 and 2%, respectively [3].

Furthermore, the appearance of new colonic lesions within 2 to 4 years after the incidence of *S. bovis/gallolyticus *bacteremia/endocarditis provides clearer evidence that *S. bovis/gallolyticus *is not merely a consequence of the tumor lesion [86]. For this reason, patients with infectious endocarditis and normal colonoscopy may be included in the group that presents risk for developing colonic cancer because of the late appearance of such lesions after the infectious episode of *S. bovis/gallolyticus*.

In terms of pathogenesis, as *S. bovis/gallolyticus *is a transient normal flora in the gut, researchers have postulated that the increased load of *S. bovis/gallolyticus *in colon might be responsible for its association with colon cancer. Several studies showed increased stool carriage of *S. bovis/gallolyticus *in patients with inflammatory bowel diseases or malignant/premalignant lesions of the colon; around 56% of patients with *S. bovis/gallolyticus *bacteremia/endocarditis showed increased faecal carriage, when compared to normal subjects or patients with benign diseases of the colon, such as colonic diverticulosis, inflammatory bowel disease, cecal volvulus, perirectal abscess and hemorrhoids (10-23%) [2,67,75].

Another clue supporting the etiological role of *S. bovis/gallolyticus*, patients diagnosed with colon cancer have only 3-6% chance to develop *S. bovis/gallolyticus *bacteremia/endocarditis [87]; this is far lower than the percentage of the detection of colorectal cancer in patients with *S. bovis/gallolyticus *bacteremia/endocarditis, >70%.

*S. bovis/gallolyticus *is shown to have indiscriminate pathogenic factors. It can uniquely colonize the thrombin of platelets and fibrin where colonies become developed with protection from new layers of platelets and fibrin that are formed by stimulation from thromboplastin; hence, *S. bovis/gallolyticus *can penetrate into the bloodstream through epithelial, oropharyngeal, dermal, respiratory, gastrointestinal, or urogenital lesions [88]. On the other hand, the ulceration of neoplastic lesions are found to be unable to form a consistent pathway for the gut microorganisms to enter the bloodstream [7]. The access of *S. bovis/gallolyticus *into blood circulation does not explain the cases of patients with infectious endocarditis and non-ulcerated colonic polyps [81].

Above all, *S. bovis/gallolyticus *bacteria were found to be actively engaged in triggering severe inflammatory reaction in colorectal mucosa, inducing inflammatory and angiogenic cytokines leading to the formation of free radicals that are implicated in the development or propagation of all types of human cancers [27,29,37,39,40,89].

Accordingly, too many clues were found supporting the etiological role of *S. bovis/gallolyticus *in the development of colorectal tumors; therefore, it is very difficult to assume a non-etiological role of these bacteria. Hence, a more detailed overview is needed to clarify the underlying mechanisms that could be pursued by *S. bovis/gallolyticus *for the etiology or propagation of colorectal tumors.

## The hypothesized mechanisms of the etiological association of *S. bovis/gallolyticus *with colorectal tumors

The other big question in the current topic, what mechanisms *S. bovis/gallolyticus *undertakes to induce, promote, or/and progress the development of neoplastic lesions. The most possible mechanisms are as follows:

### Carcinogenesis via cytokine-dependent inflammation

Chronic inflammation is associated with many malignant changes. Host genetic polymorphisms of the adaptive and innate immune response play an important role in bacteria-induced cancer formation [90-92]. Therefore, studying the immunological responses to chronic bacterial infections yields important clues on the carcinogenic mechanisms of bacterial persistent infections and clarifies the relationship between inflammation and cancer [93,94]. Clinical studies have shown that the use of non-steroidal anti-inflammatory drugs is associated with reduced risk of gastrointestinal cancers [95]; hence, these studies provide evidence on the role of inflammation in the development of gastrointestinal cancers.

**Figure 1:**
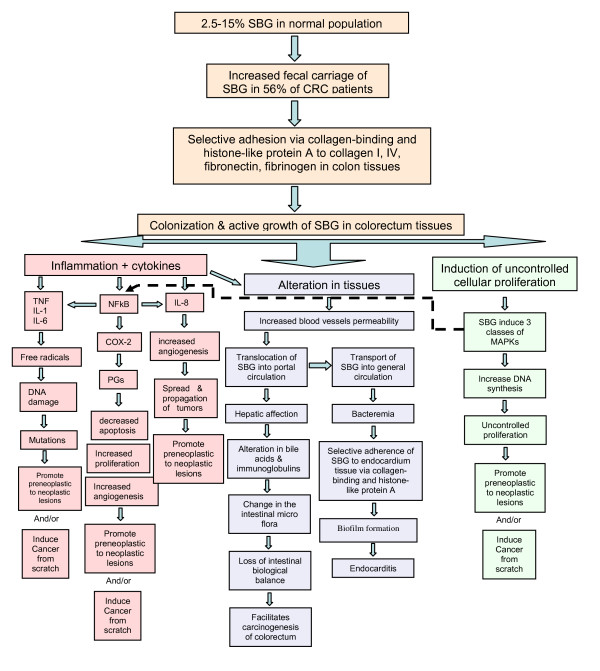
**Illustration for the discovered and suggested mechanisms underlying the etiological association of *S. bovis/gallolyticus *(SBG) bacteria with promoting, propagating, or initiating colorectal tumors, bacteremia, and endocarditis**.

*In vitro *experiments showed that the binding of *S. bovis *wall extracted antigens to various cell lines, including human colonic cancer cells (Caco-2), stimulated the production of inflammatory cytokines by those cells [38,96]. In other studies, the production of inflammatory cytokines in response to *S. bovis/gallolyticus*, such as TNF-α, IL-1β, IL-6, and IL-8, is found to contribute to the normal defense mechanisms of the host [89,97] leading to the formation of nitric oxide and free radicals such as superoxide, peroxynitrites, hydroxyl radicals, and alkylperoxy radicals [96,98]. Owing to their potent mutagenicity, all these molecular species can contribute to the neoplastic processes by modifying cellular DNA (Figure 1). On the other hand, the production of angiogenic factors in colonic mucosa, such as IL-8, which can be triggered by *S. bovis/gallolyticus *antigens, may also favor the progression of colon carcinogenesis [39,40,89,99,100] (Figure 1). This resembles *H. pylori *infection for the development of chronic inflammation in the gastric mucosa [101]. Therefore, chronic infection and subsequent chronic inflammation seem responsible for the maintenance and development of pre-existing neoplastic lesions [39,40,102].

Moreover, it was found that wall extracted antigens of *S. bovis *induced *in vitro *overexpression of cyclooxygenase-2 (COX-2) [38,96]. COX-2, via prostaglandins, promotes cellular proliferation and angiogenesis and inhibits apoptosis (Figure 1); thus it acts as a promoter in cancer pathway [103]. It is noteworthy to mention that non-steroidal anti-inflammatory drugs decrease the relative risk of gastrointestinal carcinomas through inhibiting the activity of COX-2 which is over-expressed in up to 85% of colorectal adenocarcinomas [104]. Alike, Haqqani et al., [105] revealed that the activation of leukocytes by *S. bovis/gallolyticus *releases various other inflammatory mediators (NO, free radicals, peroxynitriles, etc.) which could interfere directly or indirectly with the cell proliferation process.

The recent studies conducted by our team revealed that *S. gallolyticus *is remarkably associated with colorectal cancer and adenoma when compared to the more dominant intestinal bacteria, *B. fragilis*. This provided evidence for a possible important role of *S. gallolyticus *in the carcinogenesis of colorectal cancer from pre-malignant polyps. In addition, we found that NF-κB and IL-8 rather than other transformation factors, p21, p27 and p53 acted as highly important mediators for the *S. gallolyticus*- associated progression of colorectal adenoma to carcinoma [39]. And NF-κB most probably exerts a promoting carcinogenic effect while IL-8 exerts an angiogenic/propagating effect on colorectal mucosal cells [39]. In addition, a more recent study done by our team showed a direct and active role of *S. bovis/gallolyticus *in colonizing colorectal cancer tissues leading to the development of colorectal cancer through inflammation-based sequel via, but not limited to, IL-1, COX-2, and IL-8 [40].

Another aspect of inflammatory cytokines, the local action of cytokines or of chemical mediators is able to promote vasodilatation and the enhancement of capillary permeability, which in turn was found to support the bacterial entry at tumor sites, and increase bacterial adherence to various cells [38,89]. It has been suggested that alteration in local conditions and disruption of capillary channels at the site of neoplasm allowed *S. bovis/gallolyticus *to proliferate and gain entry into blood stream [37,38,40,96]. Therefore, *S. bovis/gallolyticus *shows characteristic potential in inducing mucosal inflammation and changing the mucosal microclimate leading most probably to tumor development and increased permeability of blood vessels which facilitates this bacterium to enter blood circulation causing bacteremia and/or endocarditits.

### Characteristic adherence potential

Members of the *S. bovis/gallolyticus *group are frequent colonizers of the intestinal tract as well as endocardial tissues. However, their ability to adhere to and colonize host tissues was largely unknown. Sillanpaa et al., [106] found recently that *S. bovis/gallolyticus *bacteria possess collagen-binding proteins and pili responsible for adhesion to colorectal mucosa as well as to endocardium (Figure 1). On the other hand, Boleij et al., [107] found a histone-like protein A on the cell wall of *S. gallolyticus *able to bind heparan sulfate proteoglycans at the colon tumor cell surface during the first stages of infection. This protein is believed to be largely responsible for the selective adhesive potential of *S. bovis/gallolyticus*. In addition, Vollmer et al. [108]found recently that the adherence of *S. bovis/gallolyticus *to the extracellular matrix proteins, collagen I, II and IV, revealed the highest values, followed by fibrinogen, tenascin and laminin. Moreover, all tested strains showed the capability to adhere to polystyrole surfaces and form biofilms [108]. Another study which assessed 17 endocarditis-derived human isolates, identified 15 *S. gallolyticus *subspecies *gallolyticus*, one *S. gallolyticus *subspecies *pasteurianus *(biotype II/2) and *one S. infantarius *subspecies *coli *(biotype II/1) for their in vitro adherence to components of the extracellular matrix. They found that *S. gallolyticus *subspecies *gallolyticus *has very efficient adherence characteristics to the host extracellular matrix; this bacteria showed powerful adherence to collagen type I and type IV, fibrinogen, collagen type V, and fibronectin [109] (Figure 1). These adherence criteria make *S. gallolyticus *subspecies *gallolyticus *a successful colonizer in both intestinal and cardiac tissues. Therefore, it has been stated that the relationship between *S. bovis/gallolyticus *endocarditis and *S. bovis/gallolyticus *colonic tumors suggests the existence of certain adhesins on the cell wall of these bacteria allowing the colonization of both colonic and vascular tissues [106,107].

### Altering the profile of bacterial flora

The members of gut microflora contribute to several intestinal functions, including the development of mucosal immune system, the absorption of complex macromolecules, the synthesis of amino acids and vitamins, and the protection against pathogenic microorganisms. In order to keep the mutual relationship between the microflora and the intestinal function, it is important that microflora is continuously kept under control to preserve gut homeostasis. When this is not achieved or perturbed, several immune disorders can arise, like allergies, inflammation, and cancer [110,111]. Increased incidence of hepatic dysfunction was reported among patients with infectious endocarditis caused by *S. bovis/gallolyticus *[77]. Both colonic pathology and liver dysfunction were determined in 92 patients with *S. bovis *endocarditis/bacteremia. Colonic pathology was identified in 51%, and liver disease or dysfunction was documented in 56% of patients with *S. bovis/gallolyticus *endocarditis/bacteremia [4]. It was conceived that either the underlying colonic disease or the alterations in hepatic secretion of bile salts or immunoglobulins may promote the overgrowth of *S. bovis *and its translocation from the intestinal lumen into the portal venous system [4] (Figure 1).

Alike, it has been speculated that *S. bovis/gallolyticus *affects portal circulation through bacterial translocation, thereby determining hepatic alterations. Modifications in the hepatic secretion of bile salts and the production of immunoglobulins contribute towards increasing the participation of *S. bovis/gallolyticus *in abnormal changes in the bacterial flora of the colonic lumen which might then promote carcinogenesis of the intestinal mucosa [7,84].

### Promoter of early preneoplastic lesions

A series of interesting experiments was conducted to investigate the role of *S. bovis/gallolyticus *in the initiation versus the propagation of colorectal cancer. Chemical carcinomas of colon were induced by giving adult rats intraperitonial injections of azoxymethane (15 mg/kg body weight) once per week for 2 weeks. Fifteen days (week 4) after the last injection of the carcinogen, the rats received, by gavage twice per week during 5 weeks, either *S. bovis *(10^10 ^bacteria) or its wall-extracted antigens (100 μg). One week after the last gavage (week 10), it was found that administration of either *S. bovis *or its antigens promoted the progression of preneoplastic lesions, but not normal tissue, into neoplastic lesions through the increased formation of hyperproliferative aberrant colonic crypts, which enhanced the expression of proliferation markers and increased the production of IL-8 in the colonic mucosa [38,89] (Figure 1). Therefore, it was suggested that *S. bovis/gallolyticus *acts as a potential promoter of early preneoplastic lesions in the colon of rats, and their cell wall proteins are more potent inducers of neoplastic transformation than the intact bacteria. Moreover, the development of colonic adenomas was increased remarkably in 50% of the tested rats together with the proliferation markers, namely the polyamine content and the proliferating cell nuclear antigen PCNA [37,38,96]. This provided extra evidence that *S. bovis/gallolyticus *acts more likely as promoter/propagator of colorectal carcinoma rather than just a consequence of the tumor lesion. However, these studies might suggest that bacteria are not sufficient to induce cancer by their own. Hence, tumor development might require independent mutations in the oncogenic signaling pathways together with chronic inflammatory conditions which are needed to promote, propagate, and spread tumor lesions [88].

### Induction of uncontrolled cellular proliferation

In the presence of wall extracted proteins of *S. bovis/gallolyticus*, Caco-2 cells exhibited enhanced phosphorylation of 3 classes of mitogen activated protein kinases (MAPKs) [38]. Several reports showed that MAPKs activation stimulates cells to undergo DNA synthesis and cellular uncontrolled proliferation [112-114] (Figure 1). Therefore *S. bovis/gallolyticus *proteins could promote cell proliferation by triggering MAPKs which might increase the incidence of cell transformation and the rate of genetic mutations. Furthermore, MAPKs, particularly p38 MAPK, can induce COX-2 which is an important factor in tumorogenesis [29,115] up-regulating the expression of NFkB which is considered the central link between inflammation and carcinogenesis, namely, inflammation-induced tumor progression [92].

### Colonization of Streptococcus gallolyticus in colorectal mucosa

The association of *S. bovis/gallolyticus *with colorectal cancer has usually been described through the incidence of *S. bovis/gallolyticus *bacteremia and/or endocarditis [1-4,44]. On the other hand, little bacteriological research has been done [116,117] on elucidating the colonization of *S. bovis/gallolyticus *in tumor lesions of colorectal cancer to confirm or refute, on solid bases, the direct link between colorectal cancer and *S. bovis/gallolyticus*. Previous studies [116,117] did not find clear evidence for the colonization of *S. bovis/gallolyticus *in colorectal tumors. This might be attributed to the complete reliance on bacteriological methods rather than more sensitive molecular assays for the detection of *S. bovis/gallolyticus *nucleic acids.

A recent study done by our team assessed the colonization of *S. bovis/gallolyticus *in the colon [40]. In this study, *S. bovis/gallolyticus*-specific primers and probes were used in PCR and in situ hybridization (ISH) assays, respectively, along with bacteriological isolation of *S. bovis/gallolyticus *to detect/isolate *S. bovis/gallolyticus *DNA/cells from feces, tumor mucosal surfaces, and from inside tumor lesions. *S. bovis/gallolyticus *was remarkably isolated, via bacteriological assays, from tumor tissues of colorectal cancer patients with history of bacteremia, 20.5%, and without history of bacteremia, 12.8%, while only 2% of normal tissues of age- and sex- matched control subjects revealed colonization of *S. bovis/gallolyticus*. On the other hand, the positive detection of *S. bovis/gallolyticus *DNA, via PCR and ISH assays, in tumor tissues of colorectal cancer patients with history of bacteremia, 48.7 and 46.1%, and without history of bacteremia, 32.7 and 28.8%, was remarkably higher than in normal tissues of controls, 4%, and 2%, respectively. In addition, by using absolute quantitative PCR for *S. bovis/gallolyticus *DNA, the *S. bovis/gallolyticus *count, in terms of copy number (CN), in tumor tissues of colorectal cancer patients with history of bacteremia, 2.96-4.72 log_10 _CN/g, and without history of bacteremia, 2.16-2.92 log_10 _CN/g, was higher than the near-zero colonization in normal tissues. Moreover, the level of *S.bovis/gallolyticus *colonization in colorectal cancer patients with history of bacteremia was found significantly higher than in colorectal cancer patients without history of bacteremia (Figure 1). This study provided several new clues. First, *S. bovis/gallolyticus *colonizes actively the lesion tissues of colorectal cancer patients rather than normal mucosal tissues. Second, the colonization of *S. bovis/gallolyticus *is mainly found inside tumor lesions rather than on mucosal surfaces. Third, the titer of the colonizing *S. bovis/gallolyticus *in colorectal cancer patients with history of bacteremia/endocarditis is much higher than in patients without history of bacteremia/endocarditis; this explains why some colorectal cancer patients develop concomitant bacteremia/*endocarditis *while others do not. Actually, the newly found selective colonization of *S. bovis/gallolyticus *explains the conclusions of an earlier report [118] stating that colonic lesions provide a suitable microenvironment for *S. bovis/gallolyticus *colonization resulting in silent tumor-associated infections that only become apparent when cancer patients become immunocompromised, as in bacteraemia, or have coincidental cardiac valve lesions and develop endocarditis. An earlier study conducted by Swidsinski team [119] found similar results to our study [40] but on different bacteria. They quantified bacteria in colonic biopsy specimens of normal and cancer patients by polymerase chain reaction and found that the colonic mucosa of patients with colorectal carcinoma but not normal colonic mucosa was colonized by intracellular *Escherichia coli*.

## Early detection of colorectal cancer by detecting *S. bovis/gallolyticus *as one of the potential causative agents

About 65% of population with age more than 60 years are at high risk for colorectal cancer which indicates the need for a proper screening test for the early detection of colorectal cancer [120]. For localized cancers, the five-year survival rate is approximately 90 percent for colon cancer and 80 percent for cancer of the rectum; this actually provides the suitable basis for improving patients' survival by applying reliable and early detection methods [30].

Very few studies were conducted to investigate the seroprevalence of *S. bovis/gallolyticus *among colorectal cancer patients. Seroprevalence of *S. bovis/gallolyticus *is considered as a candidate practical marker for the early prediction of an underlying bowel lesion at high risk population. It has been suggested that the presence of antibodies to *S. bovis/gallolyticus *antigens or the antigens themselves in the bloodstream may act as markers for the carcinogenesis in the colon [84,87,116]. In a study [121], it was stated that it might be possible to develop a test to screen patients for the presence of colonic cancer by measuring IgG antibody titer of *S. bovis/gallolyticus*. Moreover, the same report [121] revealed that there is a need for a good screening test for colonic cancer, particularly a test which could detect early lesions. The serology-based detection of colorectal cancer has advantages on other tests such as fecal occult blood which is neither sensitive nor specific or carcinoembryonic antigen which is regularly detectable in only advanced diseases [103].

Panwalker [122] revealed that the lack of any consistent difference in IgM antibody titer of *S. bovis *biotype I between colorectal cancer patients and control population suggests that the increased immune stimulation of colorectal cancer patients towards *S. bovis *occurs over a long period of time. Hence, since the association between slow evolving bacterial inflammation and colorectal cancer takes long time, it is prudent to seek specifically for IgG antibodies. Furthermore, IgG antibodies reflect an image of the past as well as the current presence of *S. bovis/gallolyticus *antigens in the circulation.

Some recent studies showed the possibility of constructing a serology test for the detection of colonic cancer based on the detection of antibody to *S. bovis/gallolyticus *or *Enterococcus faecalis *[39,123]. Therefore, a simple ELISA test with no more than 2 ml of patient's blood might be a good candidate for screening high risk individuals for the presence of premalignant neoplastic polyps, adenomas, and cancers. However, some older studies of antibody response to *S. bovis/gallolyticus *and other streptococci have found that antibody is detectable in endocarditis but not in either clinically insignificant bacteremias [124], or colonic cancers [125] by using immunoblotting, immunoflourescence and other techniques.

In a recent study of our team [39], the level of IgG antibodies, measured via ELISA, against *S. gallolyticus *subspecies *gallolyticus *was found to be significantly higher in colorectal cancer patients than in control subjects. This is in full agreement with the study of Darjee and Gibb [121] who showed that patients with colonic cancer had higher median IgG antibody titers to *S. bovis *and *E. faecalis *preparations than did the control samples. Hence, the seroprevalence of IgG antibodies against *S. gallolyticus *subspecies *gallolyticus *showed the same behavior to that against *S. bovis *biotype I NCTC8133 [121].

A question might be asked, is it reliable to consider the seroprevalence of IgG antibodies against *S. bovis/gallolyticus *as an indicator for the detection of colorectal cancer given that *S. bovis/gallolyticus *is a member of intestinal microflora in 2.5 to 15% of normal individuals. In fact there are many factors support the concept of using the seroprevalence of *S. bovis/gallolyticus *as a detection tool. First, it was shown that the fecal carriage of *S. bovis/gallolyticus *increases in cases of colorectal cancer [2,67,75]. Second, *S. bovis/gallolyticus *has showed selective adhesion characteristics to the tumor tissue of colorectum [106,107]. Third, the alteration in local conditions and the disruption of capillary channels at the site of neoplasm allow *S. bovis/gallolyticus *to proliferate and gain entry into the blood stream, [38] which ultimately induces immune system to actively produce remarkable specific antibodies towards *S. bovis/gallolyticus*. Fourth, *S. bovis/gallolyticus *was shown to colonize tumor lesions selectively at high titers and this colonization is located deeply inside tumor tissues rather than superficially on mucosal surfaces; this feature increases the chances of triggering the systemic, along with mucosal, immune response leading to the development of anti- *S. bovis/gallolyticus *IgM and IgG antibodies [40]. Fifth, biochemical tests are not helpful diagnostic tools because of the wide variety of phenotypes seen in the *S. bovis/gallolyticus *complex; thus, instead, it is necessary to use serological or molecular methods [126].

## Conclusions

It is concluded from the lump of research done in this field that *S. bovis/gallolyticus *association with colorectal tumors seems to be of etiological nature. And the pro-inflammatory potential of *S. bovis/gallolyticus *and their pro-carcinogenic properties including the leucocytic recruitment driven by *S. bovis/gallolyticus*, the tumor tissue- selective adhesion potential of *S. bovis/gallolyticus*, the selective colonization of *S. bovis/gallolyticus *in tumor cells, the suitable microenvironment of tumor tissues for the *S. bovis/gallolyticus *proliferation, the local disruption of tumor tissues and capillaries which allow the entry of *S. bovis/gallolyticus *into blood circulation, and the *S. bovis/gallolyticus*- induced cytokines and transcriptional factors, such as IL-1, IFN-γ, IL-8, and NFkB, all collectively provide evidence that *S. bovis/gallolyticus *is most probably responsible for a slow progressing carcinogenesis of colorectal mucosal tissues. Moreover, the *S. bovis/gallolyticus*- based carcinogenesis appears to occur through the transformation process from normal tissue to premalignant lesions, adenomas, to finally malignant cancerous tissues. And the proposed carcinogenic potential of *S. bovis/gallolyticus *is most likely a propagating factor for premalignant tissues. On the other hand, the early detection of colorectal adenomas or carcinomas via detection of *S. bovis/gallolyticus *DNA or their specific IgG antibodies might be of high value in screening high risk groups for colorectal cancer.

## Competing interests

The authors declare that they have no competing interests.

## Authors' contributions

AS and RR prepared the review data, collected the related references, analyzed the studied data and prior studies. AS, RR, and FAB drafted the review and prepared the review structure. all authors read and approved the final manuscript.
